# Tuberculous Chronic Prostatitis: a Neglected Disease

**DOI:** 10.1590/S1677-5538.IBJU.2025.0521

**Published:** 2025-09-25

**Authors:** André Avarese Figueiredo, Augusto de Azevedo Barreto, Victor Silvestre Soares Fanni

**Affiliations:** 1 Universidade Federal de Juiz de Fora Núcleo Interdisciplnar de Pesquisa em Urologia Juiz de Fora MG Brasil Núcleo Interdisciplnar de Pesquisa em Urologia - NIPU, Universidade Federal de Juiz de Fora - UFJF, Juiz de Fora, MG, Brasil; 2 Universidade Federal de Juiz de Fora Departamento de Cirurgia Juiz de Fora MG Brasil Departamento de Cirurgia, Universidade Federal de Juiz de Fora - UFJF, Juiz de Fora, MG, Brasil

To the editor,

An interesting and timely review on prostatitis was recently published in JAMA by Borget et al. (1). However, an important omission must be highlighted: tuberculous chronic prostatitis. In the review, tuberculosis is mentioned only once, under the "Epidemiology and Risk Factors" section of chronic bacterial prostatitis: "…Risk factors for chronic bacterial prostatitis include prior acute bacterial prostatitis, urethral surgery or catheterization, urinary stasis, unprotected anal intercourse, and genitourinary tuberculosis…." This limited reference has also been observed in other reviews and guidelines, underscoring that tuberculous chronic prostatitis remains a neglected condition (2).

Nearly 90% of new tuberculosis cases occur in 30 countries, including Brazil and 29 countries in Africa and Asia. Nevertheless, due to migration and globalization, tuberculosis must be regarded as a worldwide health concern. Urogenital tuberculosis, and specifically prostatic involvement, though uncommon in developed countries, has been documented globally (3). Importantly, *Mycobacterium tuberculosis* is a treatable cause of chronic prostatitis and is underdiagnosed rather than rare.

In Russia, Kulchavenya et al. followed a cohort of 73 patients with chronic prostatitis for at least two years, diagnosing tuberculous prostatitis in 17 patients (23.3%). This included 2 cases (11.8%) initially classified as nonbacterial chronic prostatitis and 15 cases (88.2%) classified as bacterial chronic prostatitis (4). More recently, our group in Brazil published in this journal a qualitative study analyzing 18 patients with prostatic tuberculosis (5). In 10 patients (55.6%), the presentation was chronic prostatitis, either recurrent (2 patients) or persistent with sterile pyuria (8 patients). All patients achieved pain resolution with pharmacological treatment. Notably, 6 patients were diagnosed within one year, coinciding with the implementation of systematic tuberculosis screening for all chronic prostatitis cases.

Chronic prostatitis/chronic pelvic pain syndrome (CP/CPPS) substantially impairs quality of life and remains therapeutically challenging. However, CP/CPPS is also a clinical manifestation of prostatic tuberculosis and may be effectively treated when recognized. Therefore, reviews and guidelines on prostatitis should emphasize that:

- Patients with CP/CPPS must be systematically screened for tuberculosis using culture and nucleic acid amplification testing of urine and semen.

The Authors

## Data Availability

Uninformed
